# Mucopolysaccharidosis type VI: case report with first neonatal presentation with ascites fetalis and rapidly progressive cardiac manifestation

**DOI:** 10.1186/s12881-020-0972-y

**Published:** 2020-02-19

**Authors:** Rachel Sayuri Honjo, Evelyn Cristina Nuñez Vaca, Gabriela Nunes Leal, Deipara Monteiro Abellan, Nana Miura Ikari, Marcelo Biscegli Jatene, Ana Maria Martins, Chong Ae Kim

**Affiliations:** 1 Unidade de Genética do Instituto da Criança – Hospital das Clinicas HCFMUSP, Faculdade de Medicina, Universidade de Sao Paulo, Av. Dr. Enéas Carvalho de Aguiar, 647, São Paulo, CEP 05403-000 Brazil; 2 Setor de Ecocardiografia do SADT do Instituto da Criança – Hospital das Clinicas HCFMUSP, Faculdade de Medicina, Universidade de Sao Paulo, Sao Paulo, Brazil; 3 Departamento de Pediatria - Instituto da Criança – Hospital das Clinicas HCFMUSP, Faculdade de Medicina, Universidade de Sao Paulo, Sao Paulo, Brazil; 4 Unidade de Cardiologia Pediátrica do Incor – Hospital das Clinicas HCFMUSP, Faculdade de Medicina, Universidade de Sao Paulo, Sao Paulo, Brazil; 5 Unidade Cirúrgica Infantil do Instituto do Coração – Hospital das Clinicas HCFMUSP, Faculdade de Medicina, Universidade de Sao Paulo, Sao Paulo, Brazil; 6 Departamento de Pediatria – Centro de Referência em Erros Inatos do Metabolismo, Universidade Federal de São Paulo, São Paulo, Brazil

**Keywords:** Mucopolysaccharidosis, Mucopolysaccharidosis type VI, Fetal ascites, Valvular disease, Inborn error of metabolism, Lysosomal disorder

## Abstract

**Background:**

The Mucopolysaccharidosis type VI (MPS VI), also known as Maroteaux-Lamy syndrome (OMIM 253200) is an autosomal recessive lysosomal disorder, caused by the deficiency of the enzyme N-acetylgalactosamine 4-sulfatase (also known as arylsulfatase B) due to mutations of the *ARSB* gene. Cardiologic features are well recognized, and are always present in MPS VI patients. Generally, the onset and the progression of the cardiologic symptoms are insidious, and just a few patients have developed a rapidly progressive disease. Cardiac involvement in MPS VI is a common and progressive feature. For MPS patients, cardiac evaluations are recommended every 1 to 2 years, including blood pressure measurement, electrocardiography and echocardiography. However, congestive heart failure and valvular surgical repair are not frequently seen, and if so, they are performed in adults. Here we report on an atypical MPS VI case with ascites fetalis and a rapidly progressive cardiac disease.

**Case presentation:**

A 6-month-old Brazilian male, only child of a Brazilian healthy non-consanguineous couple. During pregnancy, second trimester ultrasonography observed fetal ascites and bilateral hydrocele. Physical exam at 6 months-old revealed a typical gibbus deformity and MPS was suspected. Biochemical investigation revealed a diagnosis of MPS type VI, confirmed by molecular test. Baseline echocardiogram revealed discrete tricuspid regurgitation and a thickened mitral valve with posterior leaflet prolapse, causing moderate to severe regurgitation. The patient evolved with mitral insufficiency and congestive heart failure, eventually requiring surgical repair by the first year of age.

**Conclusions:**

We report the first case of MPS VI whose manifestations started in the prenatal period with fetal ascites, with severe cardiac valvular disease that eventually required early surgical repair. Moreover, in MPS with neonatal presentation, including fetal hydrops, besides MPS I, IVA and VII, clinicians should include MPS VI in the differential diagnosis.

## Background

The Mucopolysaccharidosis type VI (MPS VI), also known as Maroteaux-Lamy syndrome (OMIM 253200) is an autosomal recessive lysosomal disorder, caused by the deficiency of the enzyme N-acetylgalactosamine 4-sulfatase (also known as arylsulfatase B) due to mutations of the *ARSB* gene [1, 2].

The incidence of MPS VI lays between 1 in 43,261 and 1 in 1,505,160 live births [3].

The French doctors Pierre Maroteaux and Maurice Lamy published the first description of MPS VI in 1963, focusing in the orthopedic features of this condition [4].

The age of onset of the symptoms varies and so does the phenotypical spectrum, from mild to severe. Sometimes the diagnosis in the mild form (also called slowly progressing) can be missed because the symptoms are attenuated. On the other hand, in the severe form (or rapidly progressing), in which the symptoms may be present at birth, usually diagnosis gets sooner than between the 2nd or 3rd birthday. Death occurs near the 2nd or 3rd decades, the majority being caused by cardiac failure [3, 5].

Azevedo et al. [6] collected data from 28 Latin American patients (majority of whom were Brazilians) and found that the mean age at diagnosis for MPS VI was 48.9 months. The typical phenotype of this syndrome is caused by the progressive deposition of glycosaminoglycan in various tissues: dysostosis multiplex with claw hands and short stature, facial dysmorphism/coarse facies, corneal clouding, enlarged visceral organs (liver, spleen), hearing loss, airway difficulties and hernias (inguinal, umbilical). Usually, there is no cognitive impairment [3].

Cardiologic features are well recognized since early 1940’s as described by Strauss [7], and are always present in MPS VI patients [6]. The left side of the heart is more severely affected than the right side, being the most frequent features mitral/aortic valve stenosis (60–90% of patients) [8], and cardiomyopathy, which are usually observed in adult age [9].

Infrequent presentations have been reported: a 5-month-old infant with MPS VI and cardiomyopathy and a 9-month-old infant with endocarditis fibroelastosis, both ending on cardiac failure [9–11].

Generally, the onset and the progression of the cardiologic symptoms are insidious, but some patients, as the two described above and the one reported here, have developed a rapidly progressive disease [10, 11].

Formerly, the cardiologic management was preferably clinical and palliative. With the development of the enzyme replacement therapy (ERT) and hematopoietic stem cell transplantation (HSCT), the range of possibilities became wider [5].

However, surgical possibilities have also been explored. Open-heart operations in patients with mucopolysaccharidoses are extremely rare because of multiple issues such as: poor life expectancy, multiple infiltrated organs (myocardial tissue included) and, especially, airway complications [12–14].

Considering all MPS types, there are less than 30 cases reported in the literature as having undergone successful cardiac surgery, approximately half was valvular surgery (12 out of 23). In this group of patients, 26% were MPS VI (6 out of 23) [9].

Here we report a case of MPS VI whose manifestations started in the prenatal period with fetal ascites, rapidly evolving with mitral insufficiency and congestive heart failure, eventually requiring surgical repair by the first year of age.

## Case presentation

Male patient, only child of a Brazilian healthy non-consanguineous couple. Second trimester ultrasonography observed fetal ascites and bilateral hydrocele. Prenatal screening for infectious diseases was negative. There was no drug abuse during pregnancy.

The child was born at term, by cesarean section, with birth weight of 3400 g (p58), length of 48 cm (p20), and OFC 35.5 cm (p79). Clinical examination showed hydrocele, diastasis recti, and unilateral clubfoot. There were no signs of hepato or splenomegaly (and abdominal ultrasound was normal at birth). Echocardiogram at 4 days of life disclosed just patent foramen ovale. The patient was evaluated by the Genetics unit. No specific diagnosis was suspected, even though mild coarsening facies was already present. Skeletal survey performed at one-month-old, due to congenital clubfoot and dysmorphisms, revealed mild proximal misshapen metacarpals and thickening of the provisional cartilage.

When the child came back to the Genetics evaluation at 6 months-old, the mother reported she had noticed a progressive growing mass in the lower back since the patient was 2 months old. This had been investigated with X-rays and MRI in one of the patient’s visit in the Emergency Unit due to respiratory symptoms. The mother also reported that the patient had been suffering of frequent upper respiratory tract infections, needing hospitalization twice for wheezing crises.

**Fig. 1:**
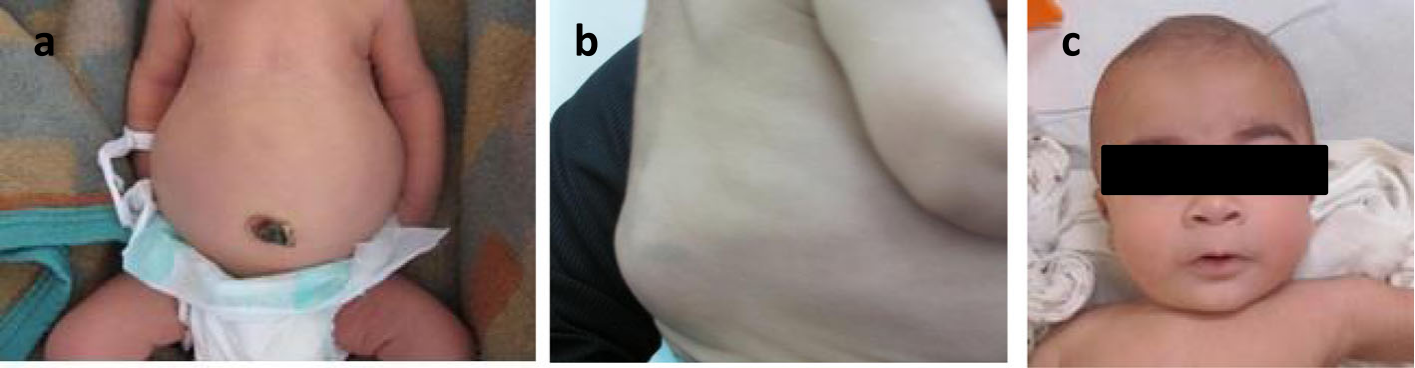
**a** Patient in the newborn period. **b** At the age of 6 months, gibbous deformity in the lumbar region. **c** At 8 months with low nasal bridge and mild coarse facies

At physical exam, the patient displayed a typical gibbus deformity (Fig. 1), which raised the suspicion of mucopolysaccharidosis. Since the patient had had fetal ascites, initially, MPS VII was suspected. Biochemical investigation revealed a diagnosis of MPS type VI (urinary glycosaminoglycans: 402 μg/mg Cr, reference value for age: 133–460 μg/mg Cr, with dermatan sulfate excretion, and enzymatic assay detected arylsulfatase B deficiency in white blood cells, with another sulfatase within the normal range).

Sequencing of the *ARSB* gene showed two pathogenic variants in *trans*: c.944G > A (p.Arg315Gln) and c.1143-1G > C.

At the age of 7 months, the patient was brought to the emergency unit due to respiratory distress. A chest X-ray showed a possible lung congestion and echocardiogram revealed discrete tricuspid regurgitation and a thickened mitral valve with posterior leaflet prolapse, causing moderate to severe regurgitation. As a result, furosemide was prescribed. Due to the rapid cardiologic changes, captopril and spironolactone were added.

**Fig. 2:**
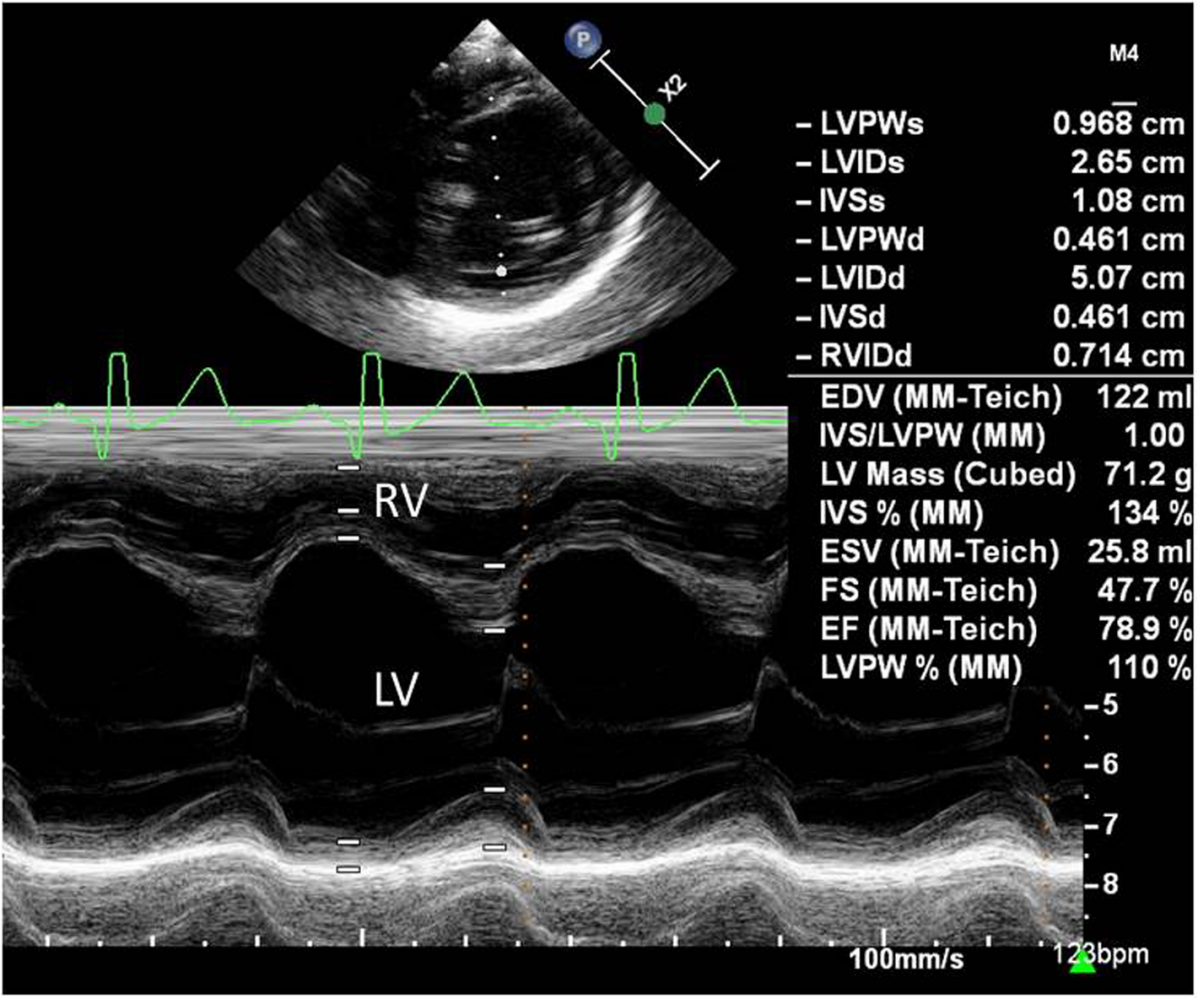
M mode of right and left ventricles (age: 9.5 months). Left ventricle is extremely enlarged (50.7 mm), with preserved ejection fraction (78.9%). RV: right ventricle; LV: left ventricle

**Fig. 3:**
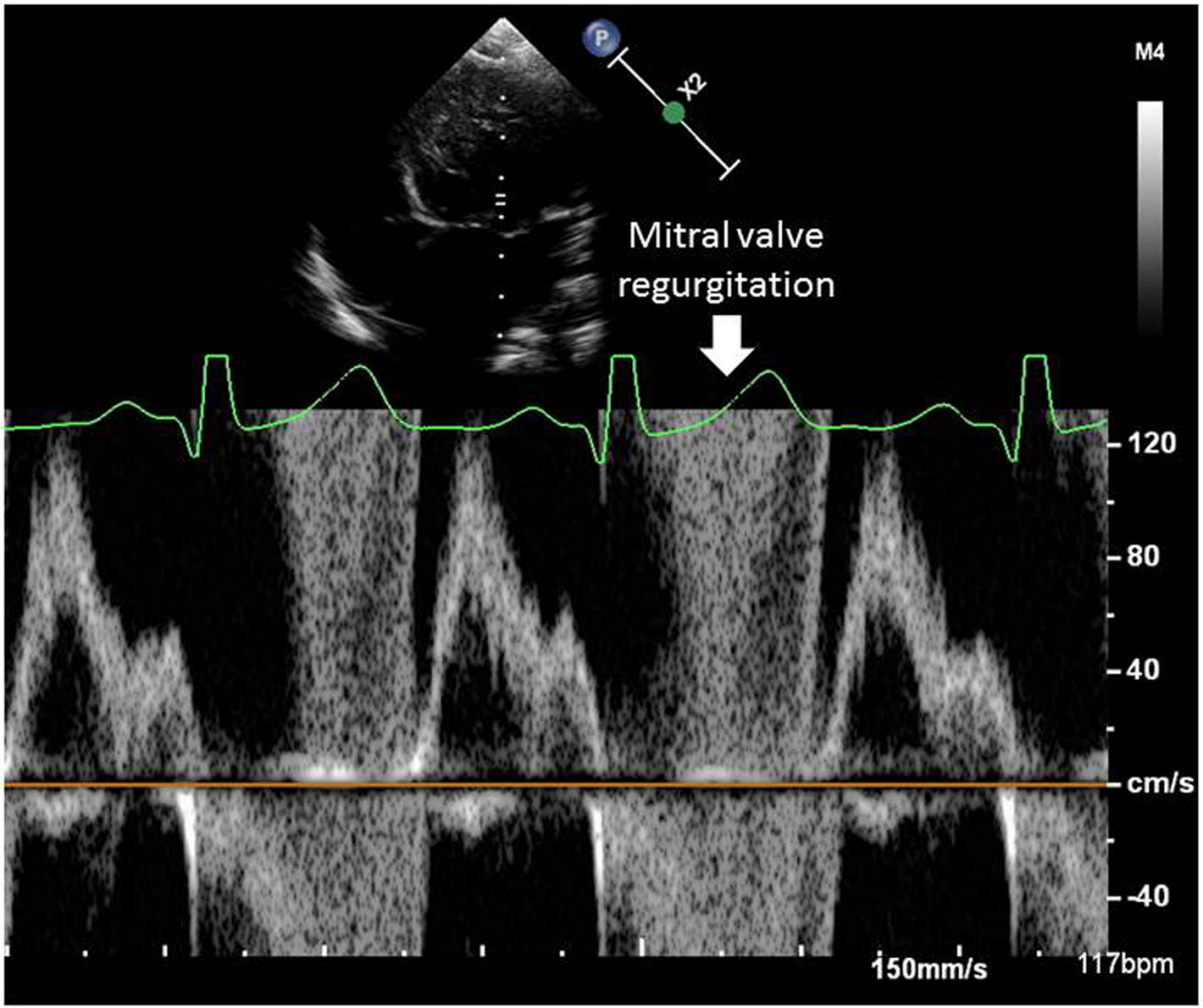
Apical 4 chamber view, focused on mitral valve. Notice the large mitral regurgitation jet on color Doppler. LV: left ventricle; LA: left atrium; MV: mitral valve

**Fig. 4:**
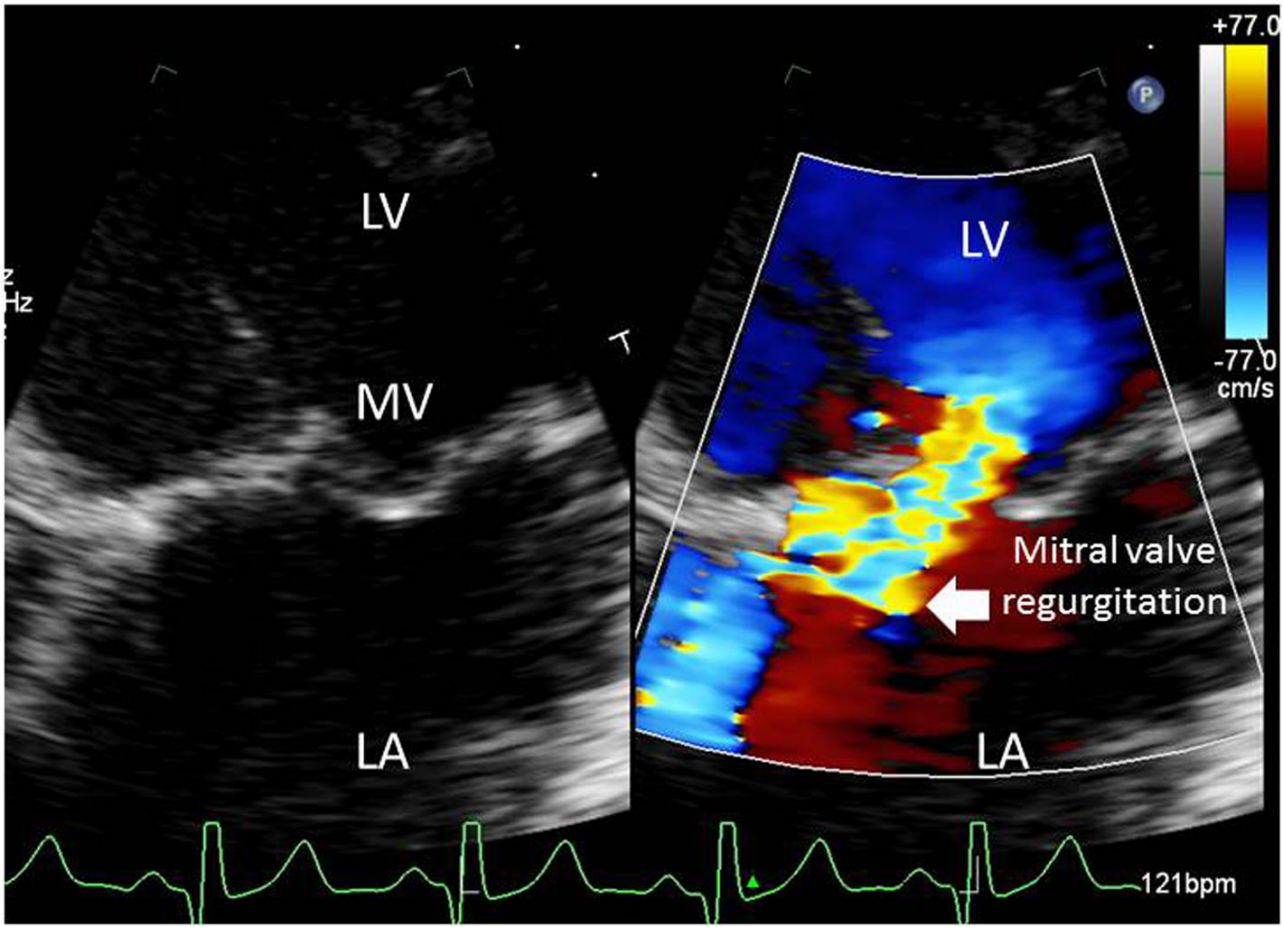
Doppler profile obtained at the mitral valve, showing severe regurgitation

At 9 months of age, the patient was hospitalized again because of cardiac decompensation. Comparative chest X-ray showed an increased cardiac area, and echocardiogram indicated worsening of mitral regurgitation. Dobutamin and dopamine were initiated, and the patient was transferred to the intensive care unit (ICU). Dobutamin was progressively withdrawn and carvedilol was introduced. Progressive improvement of respiratory distress was seen. However, a few days later echocardiogram showed left atrium and left ventricle enlargement (Fig. 2), normal left ventricular systolic function, discrete tricuspid insufficiency, mitral valve with thickened leaflets, posterior prolapsed leaflet and severe regurgitation (Fig. 3), evident with Doppler (Fig. 4).

With the rapid progression of mitral regurgitation, at 10 months the patient developed congestive heart failure and dobutamine was resumed. After stabilization, the patient was discharged receiving furosemide, captopril, spironolactone, carvedilol, digoxin, aspirin, and domperidone.

A few days after discharge, the patient was readmitted to the emergency room due to hyporexia, irritability and vomiting. The physical exam showed tachycardia, hypoxemia, respiratory distress, and hepatomegaly. He was sent to ICU, with worsening of cardiac function. The patient used bilevel positive airway pressure (BiPAP). Because of significant mitral insufficiency, left ventricular dilatation and refractory cardiac failure, in a patient with a genetic multisystemic disorder, a multidisciplinary team met to discuss the management.

**Fig. 5:**
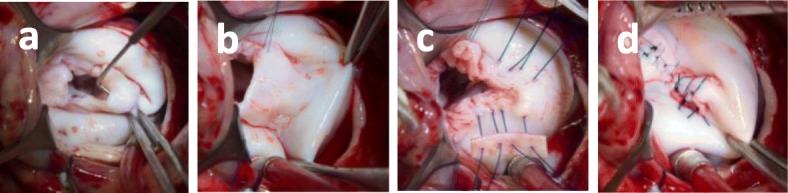
Mitral valvuloplasty, surgeon’s view: **a** Redundant mitral valve. P2 segment prolapse. **b** Quadrangular P2 resection of the mitral valve. **c** Annuloplasty with bovine pericardium patch. **d** Annuloplasty with valvuloplasty, final result

It was decided to perform cardiac surgery (valvuloplasty with mitral valve ring reduction) **(**Fig. 5**).** The patient was 11 months by the time of the surgical intervention. LV shortening fraction at 7, 9, 10 and 11 months were 70, 56, 79 and 58%, respectively. ECG showed left ventricular overload and normal sinus rhythm.

A month after the surgery, the patient began enzyme replacement therapy (ERT) with galsulfase weekly.

The last echocardiography shows discrete mitral insufficiency after valvuloplasty and last glycosaminoglycan measurement in urine was within normal range (246 μg/mg creatinine - Reference value for children under 2y: 79–256 μg/mg creatinine).

After the cardiac surgery, growth was improved (weight and height). The patient is currently with 2.5 years old and presents with mild motor delay (sat alone at 18 months and walked at 23 months old).

## Discussion and conclusions

Cardiac involvement in MPS VI is a common and progressive feature. For MPS patients, cardiac evaluations are recommended every 1 to 2 years, including blood pressure measurement, electrocardiography and echocardiography [15].

However, congestive heart failure and valvular surgical repair are not frequently seen, and if so, they are performed in adults. The mean reported age for this group of patients is 30.9 years old, ranging from 3 to 62 years [7, 9].

Table 1 shows the few MPS VI patients reported in literature who had undergone cardiac surgery (valvuloplasty or valve replacement) in spite of the high surgical risk and mortality reported for this kind of patients (20% mortality for left heart valve disease) [14, 16–18].

**Table 1:** Patients reported in the literature diagnosed with MPS VI who had undergone valvuloplasty

Characteristics			Described cases						
		Wilson et al., 1980 [16]	Tan et al., 1992 [17]				Marwick et al., 1992 [18]	Torre et al., 2016 [14]	Current case
		Male	Male	Female	Female	Female	Female	Female	Male
Age at diagnosis with MPS VI			30y	+/−31y	N/A	+/−20y	Childhood	34y	7 m
Age of onset of cardiologic symptoms		43y	28y	+/−28y	25y	+/−20y	+/− 20y	+/−37y	+/−7 m
Age at surgery		N/A	30y	34y	25y	21y	25y	40y	11 m
Pre-operative Echocardiography findings	Aortic Valve	Severe stenosis	Stenosis, with mild regurgitation	Stenosis	–	–	Mildly echo dense, with normal leaflet excursion. Minimal regurgitation	Severe stenosis, calcified cusps, moderate regurgitation	Thick valve, discrete regurgitation
	Mitral Valve	N/A	Thick and stenotic leaflets, with mild regurgitation	Stenosis	–	Severe regurgitation	Stenotic and rigid valve, commissural fusion, resembling a rheumatic valve; mild regurgitation	Thickened, with severe stenosis	Thick, redundant, prolapsed posterior cusp, central valve coaptation failure. Chordae tendon mildly thickened, a broken chordae tendon was not excluded
	Tricuspid Valve	N/A	Thick leaflets, no stenosis	–	–	Moderate regurgitation	–	–	Discrete-moderate regurgitation
	Other findings	N/A	–	–	–	Small left ventricular cavity	–	Mild left ventricular hypertrophy	Patent foramen ovale, large left auricle, Major dilatation of left ventricle
Cardiac Surgery		AVR	AVR SJA 19 mm, MVR SJA 21 mm, aortic root enlargement	AVR SJA 19 mm, MVR SJA 21 mm, aortic root enlargement	None	AVR SJA 19 mm, MVR SJA 21 mm, aortic root enlargement	MVR size 2 M Starr-Edwards 6120 prosthesis.	AVR 19 mm mechanical prosthesis.	Mitral valvuloplasty, annuloplasty with bovine pericardium patch

*N/A* Not Available, *AVR* Aortic Valve Replacement, *MVR* Mitral Valve Replacement, AVR SJA 19 mm = Aortic Valve Replacement with St. Jude aortic prosthesis, size 19 mm; MVR SJA 21 mm = Mitral Valve Replacement with inverted St. Jude aortic prosthesis, size 21 mm

In contrast with most previous reports, our patient had minimal aortic and severe mitral valvular disease with onset before the first year of life. To our knowledge, this is the second patient with MPS who has undergone successful mitral valvuloplasty; the first case being a 6-year-old boy with MPS III [19].

After valvular surgery, the patients compiled in Table 1 experienced clinical improvement with minimal residual valvulopathy or, in the worst of the cases, palliation of symptoms for several years. These outcomes may suggest that performing open cardiac surgery for selected MPS patients could be beneficial. Also, in some MPS VI cases, especially those with the rapidly progressing type, it may be important to perform an early and more frequent cardiac follow-up in case there are symptoms of cardiac etiology. Noteworthy, cardiac disease may be one of the initial signs of MPS, as reported by Fong et al. [20], who diagnosed two siblings with MPS VI with dilated cardiomyopathy and autopsy showing endocardial fibroelastosis.

Some authors show stabilization or slower deterioration of valvular disease with ERT [9, 20–23]. In our patient, an early diagnosis was also important, because even though the cardiac disease was surgically assessed, other manifestations of MPS can be treated by ERT.

Regarding our patient’s genotype, c.944G > A (p.Arg315Gln) is a common described variant, with homozygous patients showing an intermediate or severe phenotype [24, 25]. The second variant (c.1143-1G > C) is common in Spanish and Argentinian patients with MPS VI [26]; our patient’s parents did not know their ancestral origin. Our patient’s variants are related to classical MPS VI and not with the non-classical cardiac phenotype [27, 28].

Newborn screening can lead in the future to early diagnosis of MPS [29] making it possible to start ERT within the first months of age, which may prevent cardiac valve involvement and other MPS manifestations [9, 21–23].

It is the first case of MPS VI with prenatal manifestation with ascites fetalis, with a few neonatal manifestations of MPS but precocious gibbous since 2 months, and severe progressive cardiac manifestation. Fetal hydrops has been detected mainly in patients with MPS I, IVA and VII [30–32]. There is one case reported by Choy et al. in 2015 with prenatal generalized edema, necessitating intrauterine drainage of pleural effusion, and eventual biochemical diagnosis of MPS VI at 13 months of age (genotype not reported in the publication). However, this patient presented with mild to moderate valve regurgitation and progressed to severe upper cervical cord compression in the first year of life [33]. The present case indicates that, in MPS with neonatal presentation, including fetal hydrops, besides MPS VII, clinicians should include MPS VI in the differential diagnosis. This can be an extremely valuable diagnostic clue to an early diagnosis so that specific therapy and management can be implemented [34–36].

## Data Availability

For further details regarding this case report, please contact Prof Chong Ae Kim, MD, PhD (chong.kim@hc.fm.usp.br).

## Abbreviations

AVR SJA 19 mm: Aortic valve replacement with St. Jude aortic prosthesis, size 19 mm
AVR: Aortic valve replacement
BiPAP: Bilevel positive airway pressure
ERT: Enzyme replacement therapy
HSCT: Hematopoietic stem cell transplantation
ICU: Intensive care unit
LA: Left atrium
LV: Left ventricle
MPS III: Mucopolysaccharidosis type III
MPS VI: Mucopolysaccharidosis type VI
MPS VII: Mucopolysaccharidosis type VII
MPS: Mucopolysaccharidosis
MV: Mitral valve
MVR SJA 21 mm: Mitral valve replacement with inverted St. Jude aortic prosthesis, size 21 mm
MVR: Mitral valve replacement
N/A: Not available
